# Urban, semi-urban and rural difference in the prevalence of metabolic syndrome in Shaanxi province, northwestern China: a population-based survey

**DOI:** 10.1186/1471-2458-14-104

**Published:** 2014-02-01

**Authors:** Shaoyong Xu, Jie Ming, Chao Yang, Bin Gao, Yi Wan, Ying Xing, Lei Zhang, Qiuhe Ji

**Affiliations:** 1 Department of Endocrinology, Xijing Hospital, Fourth Military Medical University, 169 Changle Road West, Xi’an 710032, China; 2 Department of Oncology, Xiangyang Central Hospital, Affiliated Hospital of Hubei University of Arts and Science, Xiangyang 441021, China; 3 Department of Health Statistics, School of Preventive Medicine, Fourth Military Medical University, Xi’an 710032, China; 4 Institute of Molecular & Experimental Medicine, School of Medicine, Cardiff University, Health Park, Cardiff CF14 4XN, United Kingdom

**Keywords:** Metabolic syndrome, Prevalence, Rural, Urban, China, Cross-sectional survey

## Abstract

**Background:**

The ongoing rapid urbanization in China offers rural population opportunities not only for economic improvement but also for substantial health risks. Albeit some researches related to rural-urban difference of metabolic syndrome (MS), there lacks studies focusing on this point in undeveloped provinces in China.

**Methods:**

The survey, as part of China National Diabetes and Metabolic disorders Study, was conducted in Shaanxi province from June 2007 to May 2008. A total of 3,297 adults aged 20 years or older were included, of which 1,467 individuals were from urban areas, 839 from semi-urban areas, and 890 from rural areas. The MS was defined according to the 2009 Joint Interim Statement.

**Results:**

The age-standardized prevalence of MS was significant higher in rural residents than in urban counterparts (29.0% vs. 25.9%, *P* = 0.017), in particular among females (30.2% vs. 24.4%, *P* = 0.003). After adjusted for the listed risk factors, rural residents had a 27.6% increased risk of having MS than urban residents. With respect to MS components, the crude prevalence of raised fasting glucose and raised blood pressure was significantly greater in rural than in urban participants. However, no significant difference in the prevalence of MS was observed between semi-urban and urban participants.

**Conclusions:**

Rural residents in Shaanxi province, northwest China, were at increased risk of MS, which could be partly explained by sociodemographic and lifestyle differences. In addition, the gap between urban and semi-urban areas seemed to be minimized in related to MS prevalence. Much more attention should be paid to and intervention strategies were needed to address the rural-urban disparities in China.

## Background

Metabolic syndrome (MS) is defined as a cluster of metabolic abnormalities including abdominal obesity, hyperglycemia, hypertension, and dyslipidemia [1,2]. The patients with MS are at an increased risk of diabetes, cardiovascular disease and subsequent mortality [2-4]. With the increasing obesity and sedentary lifestyle during the past decade [5-7], the prevalence of MS has been rising worldwide [8-10]. Although it’s well documented that the MS is highly prevalent in urban residents, some epidemiological studies suggest that the rural residents are associated with higher or comparative prevalence of MS among adults in the Unites States [11], Korea [12,13] and Thai [14].

Different from the developed provinces in which urbanization has been almost achieved, Shaanxi province, as an undeveloped representative of China, is at an accelerated process of urbanization nowadays, which results in rural industrialization, conversion of farmland, and in situ “urbanized rural residents” [15,16]. The ongoing urbanization offers rural population opportunities not only for economic improvement but also for substantial health risks (air pollution, water hazard, change of diet pattern and inadequacy of activities, etc.) [15,16]. Therefore, given that the prevalence of overweight and obesity approximately doubled in rural residents over a 10-year period (1992-2002) and rural adults had a much faster annual increase than rural counterparts (2.52 vs. 0.24) [17], we hypothesized that Chinese rural adults in some places, e.g. undeveloped provinces, may experience an increased risk of MS compared with urban adults. Albeit some researches related to rural-urban difference of MS [18-22], there lacks studies focusing on this point in undeveloped provinces in China.

We utilized the data of the 2007-08 China National Diabetes and Metabolic Disorders Study, aiming to evaluate the rural-urban differences in prevalence of MS and its individual components in Shaanxi province, northwestern China.

## Methods

### Study population

This cross-sectional survey, as part of China National Diabetes and Metabolic disorders Study [23], was conducted from June 2007 to May 2008. A multistage, stratified sampling method was used to select a representative sample of persons with age 20 years or older in the general population in Shaanxi province. In the first stage, a provincial capital (Xi’an), a midsize city (Yan’an), a developed country and an undeveloped country were selected. In the second stage, two city regions from the two cities, plus two country-seats and two rural townships from the two countries were randomly selected. In the third stage, two street districts or rural villages were randomly selected from each urban city district (or country-seat) and rural township, respectively. In the final stage, individuals were randomly sampled stratified by gender and by age distribution based on the 2006 population data of Shaanxi province [24].

The study was primarily designed to provide accurate estimates of the prevalence of diabetes. A sample size of 3,200 individuals was planned to provide an 80% power to detect a 3% prevalence of diabetes [with 95% confidence intervals (95% CI), α = 0.05, β = 0.2], which was sufficient to examine MS due to its higher prevalence compared with diabetes. A total of 3,930 individuals who had lived in their residence for 5 years or longer were randomly chosen and invited. A total of 3,297 individuals completed the survey and examination (the overall response rate: 83.9%). After the exclusion of 101 individuals for whom any data of waist circumference, fasting blood glucose, blood pressure, serum triglycerides, and serum high-density lipoprotein cholesterol (HDL-c) were missing, 3,196 individuals were finally included as study participants, of which 1,467 individuals were from urban areas (street district from urban city), 839 from semi-urban areas (street district of country-seat), and 890 from rural areas (villages from rural township). The study was approved by the Ethics Committee of Xijing Hospital, Fourth Military Medical University. All the participants signed written informed consent prior to data collection.

### Data collection

A standardized questionnaire was designed and performed by trained nurses or physicians at local health stations or in community clinics to collect data on demographic characteristics, lifestyle risk factors, personal medical history, and family history of diseases [25]. Educational level was categorized as elementary school or uneducated, secondary school, and college or above. Yearly family income was classified as below 10,000 China Yuan (CNY) [1,400 US Dollar (USD)], 10,000-30,000 CNY (1,400-4,200 USD) and above 30,000 CNY (4,200 USD). Cigarette smoking was defined as a lifetime history of smoking at least 100 cigarettes. Alcohol drinking was defined as consuming alcohol at least once per week. Physical activity was defined as participating in moderate or vigorous activity for 30 minutes or more per day at least 3 days a week. Family history of disease was defined as at least one of parents, brothers or sisters diagnosed a disease in a lifetime by self-reporting. Body weight and height were measured without shoes and in light clothing, and body mass index (BMI) calculated as weight in kilograms divided by the square of height in meters. Waist circumference was measured at the middle point between the costal margin and iliac crest. Blood pressure was measured using a standardized mercury sphygmomanometer in the sitting position after at least 5 minutes of rest; two consecutive readings of blood pressure were taken on the same arm and the mean of the 2 measures was used for analysis [23].

After at least 10 hours of overnight fasting, all participants were administered an oral glucose tolerance test to measure blood glucose. A standard 75-g glucose solution was given to participants with no history of diabetes, while a steamed bun that contained approximately 80 g of complex carbohydrates was given to those with a self-reported history of diabetes for safety reasons. Blood samples were collected at 0, 30, and 120 minutes after the glucose or carbohydrate load to measure blood glucose. Fasting blood samples were also collected to measure serum triglycerides and HDL-c level with the use of an enzymatic method. All laboratory measurements met a standardization and certification program [23].

### Definition

The MS was defined according to the 2009 Joint Interim Statement (2009 JIS) proposed by the International Diabetes Federation Task Force on Epidemiology and Prevention, the National Heart, Lung, and Blood Institute, the American Heart Association, the World Heart Federation, the International Atherosclerosis Society, and the International Association for the Study of Obesity, using the Asian criteria for central obesity when ≥ 3 of the following components were present [2]: 1) larger waist circumference: ≥ 90 cm (males) or ≥ 80 cm (females); 2) raised fasting triglycerides: ≥ 1.69 mmol/l or the use of lipid medications; 3) raised blood pressure: systolic blood pressure ≥ 130 mmHg, or diastolic blood pressure ≥ 85 mmHg, or the use of antihypertensive medications; 4) raised fasting glucose: ≥ 5.6 mmol/l or the use of diabetes medications; 5) lowered HDL-c: < 1.04 mmol/l (male) or < 1.29 mmol/l (female).

### Statistical analysis

Data analysis was conducted by SPSS 18.0 or Stata 11.0 and data were expressed as mean ± standard deviation (SD), median with interquartile range, or percentage as suitable. Comparisons between groups were analyzed by *t*-test for measurement data, and chi-square test for enumeration data. *P* < 0.05 was defined as the threshold for statistical significance.

Age-standardized prevalence estimates and 95% confidence intervals (95% CI) of MS for urban, semi-urban and rural residents stratified by sex were calculated with Stata *svy* command (direct standardization) to account for the overall population. The calculations were weighted based on the 2006 population distribution of Shaanxi province [24].

Stepwise multivariate analysis was utilized to examine the association between residents’ location and MS. The reference group was urban residence. In model 0, the unadjusted odds ratio (OR) and 95% CI for MS were calculated. Model 1 was adjusted for age, sex and ethnics; Model 2 was further adjusted for sociodemographic and lifestyle variables including educational level, yearly family income, cigarette smoking, alcohol drinking and physical activities; and model 3 was added for previous factors plus family history of diabetes and hypertension.

## Results

The clinical characteristics of study participants were showed in Table 1. Rural residents were more likely to have lower education and lower level of yearly family income compared with urban residents. The proportion of alcohol drinking, soft drinks and physical activities were lower in rural residents than in urban residents. Only 9.7% of rural residents had a family history of diabetes, as compared with a percentage of 13.% in urban residents. However, there were no significant differences between semi-urban and urban residents except for ethnics, educational level, physical activities, diastolic blood pressure and serum HDL-c level.

**Table 1:** Clinical characteristics of study participants in Shaanxi province, northwestern China

**Variable**	**Urban**	**Semi-urban**	**Rural**
Number	1467	839	890
Age, years	43.8 ± 14.3	42.7 ± 13.0	44.3 ± 13.5
Sex (male), n (%)	625 (42.6)	335 (39.9)	375 (42.1)
Ethnics (Han), n (%)	1423 (98.7)	821 (99.6)*	870 (98.2)
Educational level, n (%)			
Elementary school or below	359 (24.9)	142 (17.8)*	180 (20.3)
Secondary school	613 (42.6)	335 (42.0)	602 (67.9)**
College or above	467 (32.5)	321 (40.2)*	104 (11.7)**
Yearly family income, n (%)	(n = 1266)	(n = 756)	(n = 801)
10000 CNY or below	490 (38.7)	307 (40.6)	501 (62.5)**
10000-30000 CNY	571 (45.1)	339 (44.8)	257 (32.1)**
30000 CNY or above	205 (16.2)	110 (14.6)	43 (5.4)**
Cigarette smoking, n (%)	348 (23.7)	187 (22.3)	218 (24.5)
Alcohol drinking, n (%)	393 (27.0)	210 (25.1)	166 (18.9)**
Physical activities, n (%)	568 (38.9)	375 (44.9)*	257 (29.1)**
Soft drinks, n (%)			
Once or below per week	1165 (81.7)	699 (85.3)	772 (88.0)**
2-4 times per week	159 (11.2)	72 (8.8)	83 (9.5)
5 times or above per week	102 (7.2)	48 (5.9)	22 (2.5)**
Family history of DM, n (%)	182 (13.2)	99 (12.6)	68 (9.7)**
Family history of HT, n (%)	528 (36.0)	319 (38.0)	340 (38.2)
Waist circumference, cm	81.8 ± 10.2	81.5 ± 9.9	82.7 ± 10.6**
Systolic blood pressure, mmHg	119.6 ± 18.8	118.8 ± 19.2	125.1 ± 22.1**
Diastolic blood pressure, mmHg	77.2 ± 11.3	76.2 ± 11.1*	76.3 ± 12.1**
Fasting glucose, mmol/l	5.22 ± 1.38	5.16 ± 1.34	5.58 ± 1.45**
Serum triglycerides, mmol/l	1.58 ± 1.18	1.53 ± 0.96	1.49 ± 1.03
Serum HDL-c, mmol/l	1.30 ± 0.30	1.26 ± 0.30*	1.32 ± 0.31

*semi-urban vs. urban, *P* <0.05; **rural vs. urban, *P* <0.05; CNY: China Yuan; DM: diabetes mellitus; HT: hypertension; HDL-c: high-density lipoprotein cholesterol.

The age-standardized prevalence of MS in all, male and female urban participants was 25.9%, 27.6% and 24.4%, respectively. In all and female subgroups, rural participants had a significant higher prevalence compared with urban counterparts (29.0% vs. 25.% and 30.2% vs. 24.4%, respectively). However, no significant differences in the prevalence of MS were observed between semi-urban and urban participants in all, male and female subgroups (Table 2).

**Table 2:** Crude and age-standardized prevalence of metabolic syndrome among the study participants stratified by sex in Shaanxi province, northwestern China

**Age group**	**Urban**		**Semi-urban**			**Rural**		
**year**	**n/N**	**% ± SD**	**n/N**	**% ± SD**	***P *value**	**n/N**	**% ± SD**	***P *value**
All								
20-30	21/267	7.9 ± 3.3	14/143	9.8 ± 4.9		7/148	4.7 ± 3.5	
30-40	68/374	18.2 ± 3.9	43/228	18.9 ± 5.2		47/205	22.9 ± 5.8	
40-50	101/343	29.4 ± 4.8	55/214	25.7 ± 5.9		66/199	33.2 ± 6.6	
50-60	93/228	40.8 ± 6.4	82/174	47.1 ± 7.5		112/221	50.7 ± 6.6	
≥ 60	130/255	51.0 ± 6.2	36/80	45.0 ± 11.1		63/117	53.8 ± 9.2	
Crude	413/1467	28.2 ± 2.4	230/839	27.4 ± 3.0		295/890	33.1 ± 3.1	
Standardized*		25.9 ± 2.1		24.2 ± 2.4	0.693		29.0 ± 2.7	0.017
Male								
20-30	18/112	16.1 ± 6.9	8/61	13.1 ± 8.7		3/61	4.9 ± 5.6	
30-40	47/172	27.3 ± 6.7	28/86	32.6 ± 10.1		23/85	27.1 ± 9.6	
40-50	35/127	27.6 ± 7.8	26/86	30.2 ± 9.9		22/83	26.5 ± 9.7	
50-60	35/94	37.2 ± 9.9	22/64	34.4 ± 12.0		38/96	39.6 ± 10.0	
≥ 60	46/120	38.3 ± 8.8	15/38	39.5 ± 6.3		27/50	54.0 ± 14.3	
Crude	181/625	29.0 ± 3.6	99/335	29.6 ± 5.0		113/375	30.1 ± 4.6	
Standardized*		27.6 ± 3.5		26.9 ± 3.7	0.643		27.2 ± 4.2	0.784
Female								
20-30	3/155	1.9 ± 2.2	6/82	7.3 ± 5.8		4/87	4.6 ± 4.5	
30-40	21/202	10.4 ± 4.2	15/142	10.6 ± 5.1		24/120	20.0 ± 7.3	
40-50	66/216	30.6 ± 6.1	29/128	22.7 ± 7.3		44/116	37.9 ± 9.0	
50-60	58/134	43.3 ± 8.5	60/110	54.5 ± 9.5		74/125	59.2 ± 8.7	
≥ 60	84/135	62.2 ± 8.3	21/42	50.0 ± 15.8		36/67	53.7 ± 12.3	
Crude	232/842	27.6 ± 3.0	131/504	26.0 ± 3.8		182/515	35.3 ± 4.1	
Standardized*		24.4 ± 2.4		23.6 ± 3.0	0.842		30.2 ± 3.5	0.003

*The calculations were weighted based on the 2006 population distribution of Shaanxi province. SD: standard deviation.

Comparison between rural and urban participants showed that the prevalence of raised fasting glucose and raised blood pressure was significantly greater in rural than in urban participants among all, male and female subgroups. In females, the prevalence of larger waist circumference was significant higher in rural areas. However, comparison between semi-urban and urban participants showed no significant differences in prevalence of all the individual components among all, male and female subgroups (Table 3).

**Table 3:** Crude prevalence of individual component of metabolic syndrome among the study participants stratified by sex in Shaanxi province, northwestern China

**Variable**	**Urban**		**Semi-urban**			**Rural**		
	**n/N**	**% ± SD**	**n/N**	**% ± SD**	***P *value**	**n/N**	**% ± SD**	***P *value**
All								
Raised fasting glucose	348/1467	23.7 ± 2.2	176/839	21.0 ± 2.7	0.130	355/890	39.9 ± 3.2	<0.001
Raised blood pressure	504/1467	34.4 ± 2.4	264/839	31.5 ± 3.1	0.157	386/890	43.4 ± 3.2	<0.001
Raised serum triglycerides	475/1467	32.4 ± 2.4	276/839	32.9 ± 3.2	0.799	272/890	30.6 ± 3.0	0.358
Lowered serum HDL-c	529/1467	36.1 ± 2.4	337/839	40.2 ± 3.2	0.050	304/890	34.2 ± 3.1	0.349
Larger waist circumference	603/1467	41.1 ± 2.5	344/839	41.0 ± 3.3	0.961	397/890	44.6 ± 3.3	0.095
Male								
Raised fasting glucose	139/625	22.2 ± 3.3	84/335	25.1 ± 4.6	0.322	148/375	39.5 ± 5.0	<0.001
Raised blood pressure	243/625	38.9 ± 3.9	124/335	37.0 ± 5.2	0.571	184/375	49.1 ± 5.1	<0.001
Raised serum triglycerides	247/625	39.5 ± 3.8	146/335	43.6 ± 5.3	0.223	125/375	33.3 ± 4.8	0.050
Lowered serum HDL-c	170/625	27.2 ± 3.5	100/335	29.9 ± 5.0	0.384	97/375	25.9 ± 4.5	0.645
Larger waist circumference	241/625	38.6 ± 3.9	130/335	38.8 ± 5.2	0.941	133/375	35.5 ± 4.8	0.328
Female								
Raised fasting glucose	209/842	24.8 ± 2.9	92/504	18.3 ± 3.3	0.005	207/515	40.2 ± 4.4	<0.001
Raised blood pressure	261/842	31.0 ± 3.1	140/504	27.8 ± 3.9	0.211	202/515	39.2 ± 4.2	0.002
Raised serum triglycerides	228/842	27.1 ± 3.0	130/504	25.8 ± 3.8	0.267	147/515	28.5 ± 3.9	0.343
Lowered serum HDL-c	359/842	42.6 ± 3.3	237/504	47.0 ± 4.4	0.117	207/515	40.2 ± 4.2	0.376
Larger waist circumference	362/842	43.0 ± 3.3	214/504	42.5 ± 3.3	0.848	264/515	51.3 ± 4.3	0.003

SD: standard deviation; HDL-c: high-density lipoprotein cholesterol.

The multiple logistic regression models were showed in Table 4. In unadjusted analysis (Model 0), rural participants had 1.237 times higher odds of having MS than urban counterparts (95% CI: 1.001-1.530). After adjusted for age, sex, and ethnics (Model 1), rural participants still had a significantly higher odds ratio (OR: 1.288, 95% CI: 1.028-1.613). In the next two steps, after further adjustment for educational level, yearly family income, cigarette smoking, alcohol drinking, and physical activities (Model 2) and family history of diabetes and hypertension (Model 3), respectively, the significance still existed and rural participants finally had a 27.6% increased risk of having MS than urban adults. Besides, the odds ratios of MS for semi-urban participants compared with urban counterparts showed no statistical significance in all the models.

**Table 4:** Odds ratios for metabolic syndrome among rural and semi-urban as compared to urban participants in Shaanxi province, northwestern China

**Variable**	**Urban**	**Semi-urban**				**Rural**			
	**Ref.**	***x***^***2***^	**OR**	**95% CI**	***P *value**	***x***^***2***^	**OR**	**95% CI**	***P *value**
Model 0	1.000	0.002	0.995	0.806-1.228	0.961	3.862	1.237	1.001-1.530	0.049
Model 1	1.000	0.066	1.029	0.824-1.285	0.798	4.859	1.288	1.028-1.613	0.028
Model 2	1.000	0.111	1.038	0.831-1.297	0.739	4.779	1.286	1.026-1.610	0.029
Model 3	1.000	0.088	1.034	0.829-1.293	0.767	4.419	1.276	1.017-1.601	0.036

OR: odds ratio; CI: confidence interval. The covariables in each step enter the model by a forward method. Model 0: unadjusted; Model 1: adjusted for age, sex and ethnic; Model 2: further adjusted for educational level, yearly family income, cigarette smoking, alcohol drinking and physical activities; Model 3: further adjusted for family history of diabetes and family history of hypertension.

## Discussion

In this paper, we classified study participants according to their locations as urban, semi-urban and rural residents instead of traditional dichotomy in China. The results showed that the prevalence of MS was significant higher in rural residents than in urban counterparts, in particular among females. After adjusted for the listed risk factors, rural residents had a 27.6% increased risk of having MS than urban residents. With respect to MS components, the prevalence of raised fasting glucose and raised blood pressure was significantly greater in rural than in urban participants. However, no significant difference in the prevalence of MS was observed between semi-urban and urban participants.

Unlike our findings, previous studies had reported a higher prevalence of MS in urban than in rural areas in China. For instance, Gu D. et al. performed a survey in a nationwide sample of 15,540 Chinese adults aged 35-74 years in 2000-01 and indicated that the age-standardized prevalence of MS was higher in urban (18.6%) than in rural (12.7%) residents [22]. Lao XQ et al. analyzed a provincial representative sample of 6468 residents aged 20 years or above in Guangdong province during 2002 and the results showed that the urban population had a higher prevalence of MS than the rural population (8.99% vs. 5.27%) [19]. Similar finding was also suggested in Weng X.’s [21] and Zhao J.’s [20] surveys which conducted in 2002 and 2006 in eastern China, respectively. However, we found that rural residents had a 27.6% increased risk of having MS than urban counterparts after adjustment, and raised fasting glucose and raised blood pressure were the two individual components related to the rural-urban difference of MS.

This discrepancy could be partly explained by the fact that our survey was conducted in an undeveloped province within five years while most of previous studies analyzed data from surveys in developed provinces or ten years ago. Shaanxi province, different from developed provinces in which the urbanization has been almost achieved, is at an accelerated process of urbanization. The ongoing urbanization brings rural residents both higher income and an unhealthy lifestyle, as poor early life conditions increase the risk of obesity in a subsequently more socio-economically developed environment [26]. With the expansion of city areas, rural villages are subsumed, large areas of farmlands are converted to urban use, their rural residents are compensated for several sets of housing, and some residents often turn to renting property as an alternative source of income, all of which result in the deficit of working activities in farmlands. Even residents in remote rural villages can also receive financial support from family members in urban areas, which could affect a rural family’s agricultural activities. Importantly, this deficit in energy expenditure is unlikely to be compensated for through leisure physical activities [27]. Not to mention that little awareness and knowledge of the risks of inactivity among rural residents worsen inadequacy of leisure physical activities [28]. In addition, rapid urbanization impacts on diet shifts among rural residents, including large increases in energy density, in the proportion of the population consuming a high fat diet and in animal product intake [29]. The direct consequence of inadequacy activity and diet change is the increased prevalence of obesity and metabolic abnormalities (e.g. MS, raised fasting glucose, raised blood pressure), as shown in our study. To some extent, the urbanized rural villages have shared the environmental risk factors of cities (e.g. chemical contamination of foodstuffs, water and air), but not enjoyed the advantage (e.g. available recreational resources, adequate medical insurance, and convenient access to health care services). That’s the reason rural-urban difference of MS prevalence in our study persisted even after the adjustment of demographic and lifestyle risk factors, indicating that the listed factors only partly explain and some other factors, such as environmental hazards, may contribute to the regional difference. Actually, previous epidemiological studies also indicated that rural residents may face an increased risk of MS [11-14]. For example, Trivedi T. et al. analyzed the data from the 1999-2006 Nation Health and Nutrition Examination Survey and estimated the prevalence of MS was higher in rural than in urban residents (39.9% vs. 32.8%) [11].

Different from the report by Ntandou G [30], no significant difference in the prevalence of MS was observed between semi-urban and urban areas in our study, suggesting that the gap between urban and semi-urban areas seems to be closed due to urbanization in Shaanxi province. Our findings revealed that the two areas face a similar background such as MS prevalence and lifestyle risk factors. In Shaanxi provinces, some educational institutions move to semi-urban areas, which leads to a higher educational level in semi-urban than in urban areas.

Although our study was a multistage, stratified randomly sampling survey, some limitations should be considered. Firstly, the cross-sectional natural decides a possible deficiency of causal inferences. Secondly, urban and women participants were oversampled, which may result in a potential selection bias contributed to the rural-urban difference. Thirdly, risk factors such as dietary intake (or global dietary index), environmental hazards, and diabetes or hypertension knowledge were not assessed for some reasons. We were thus unable to further explore the possible cause of rural-urban difference. Lastly, the data of yearly family income in our survey could not totally reflect the real disposable income which is hard to obtain due to privacy in China.

## Conclusions

The results of the study indicated that rural residents in Shaanxi province, northwest China, were at increased risk of MS, which could be partly explained by sociodemographic and lifestyle differences. Furthermore, the gap between urban and semi-urban areas seemed to be minimized in related to MS prevalence. It’s in question whether our study was an isolated issue or would happen in another province. However, it’s most likely that China’s urbanization will continue unabated. Considering the high prevalence of MS among rural populations and the likelihood of developing medical complications, much more attention should be paid and intervention strategies were needed to address the rural-urban disparities in China.

## Competing interests

The authors declare that they have no conflict of interests.

## Authors’ contributions

SX, JM and CY contributed equally to the study. SX, JM had full access to all the data in the study, and took responsibility for the integrity of the data and the accuracy of the analysis. QJ, SX, JM, and CY conceived the study. All authors contributed to the analysis, which was mainly done by SX and JM. SX and CY wrote the first draft, and all authors contributed to the writing of the final report. All authors read and approved the final manuscript.

## Pre-publication history

The pre-publication history for this paper can be accessed here:

http://www.biomedcentral.com/1471-2458/14/104/prepub
